# Foreign Correspondence

**Published:** 1869-04

**Authors:** 


### Foreign Correspondence.

ALLGEMEINEN KRAUKENHAUS,

*Vienna, January 26th, 1869.*

CHICAGO MEDICAL EXAMINER:—It is scarcely necessary for an American to come to Vienna to learn surgery; not only because it is well taught at home, but because it is scarcely better taught here. The student, however, can here enjoy more advantages by means of private courses, which he cannot at present in Chicago, to the same degree, but which might be more fully extended to him there. Too much cannot be said, perhaps, in praise of Prof. Billroth for his investigations and discoveries in Physiology, Pathological Histology, etc., which alone rank him among the first of men in medical science. As an operator, he is probably only to be numbered with others.

The Department of Surgery is taught by Profs. Billroth and Baron von Dumreicher, who have charge of most of the surgical wards.

Each of the wards has about eighty beds. Each of them gives a clinic an hour and a half long five days in the week, in separate auditoriums, at the same hour. Each Professor has two Assistants, who live in the hospital, and correspond to our House Physicians, except that they are paid for their service, and are allowed to perform some operations. Besides these, there are six operating pupils, who are allowed "inside the ring" for the especial purpose of fitting themselves for surgeons. They are required to pass an examination for the position, and can hold it for two years.

While, however, they enjoy this great advantage themselves, they cut off many benefits from the students. They closely surround every patient laid on the table, whether their services are needed or not, and often so obscure the operation, as to make it of little benefit to the students. I often wonder why it is not corrected.

The out-patients or ambulants are first attended to, who range in number from four to twelve daily.

Candidates for graduation are called, per list, into the ring to examine the patients, give diagnosis, prognosis, treatment, and sometimes to perform unimportant operations, as opening an abscess, etc. Each has one or more cases, according as they are trivial or important. The student is often subjected to a pretty close quiz. In this way, I presume, the Professor understands very well the attainments of each student, and his fitness for graduation at the final examination.

A similar plan introduced into our schools somewhat more generally, would tend, I should judge, to excite a somewhat more thorough study on the part of the student, as every one is more or less chagrined to make a failure before the class.

The supposition, that when young men are old enough to commence the study of medicine, no stimulus is necessary, except the vast and interesting field before them, is somewhat Utopian, as it often fails to produce well-read men; especially when the standard for graduation is as low as in many of our schools.

After the ambulants are dismissed, patients from the wards, requiring more important operations, or illustrating more complicated treatment, are brought in for operations. These number from one to four daily. After the clinic, we often look hastily through the wards. Prof. Billroth gives, also, a private course in Theoretical Surgery.

There is also a private course given by one of the Assistants on bandages, in which each student is allowed to apply the different varieties on the living subject.

Another is given on Minor Surgery, treatment of ulcers, etc.

Another on dislocations and fractures, with the applications of splints and other apparatus.

Another on Orthopœdic Surgery, which I understand is almost wholly theoretical, few or no patients being used. I may say, in this connection, that a course is also given on the medical and surgical treatment of the urinary organs; which, however, I understand, is not very valuable.

Courses are also given by the Assistants on Operative Surgery, when the student makes all the ordinary operations on the cadaver.

Prof. Patrubau, who formerly occupied the chair of Physiology, Anatomy, etc., at Prague, but lost his position in 1848, I think, and again in 1852, by joining and leading the Liberal party against the army and Government, gives a course on Topographical Anatomy, with its relations to surgical diseases. It is thought that he may regain his position, and become professor of this subject in this University.

I may add, in conclusion, that many and all of these private courses are not necessarily continuous, but ordinarily depend upon the formation of a class of ten or more individuals. Private courses, in general, here, are supported almost entirely by foreigners. Several private classes I've been in, were composed largely of Americans; and but for them, some of the courses would scarcely have been given. The majority of Austrian and Hungarian students content themselves with the regular courses.

The ordinary duration of private courses is from five to six weeks, with three to five clinics per week. For some of the courses the foreigner enjoys the privilege of paying a double price, which would seem a poor policy to attract foreign students. F.

---

I adjoin the outlines of a case of lithotrity, which I saw some time since, as illustrating what accident may happen to the most skilful:—

A young man of full habit was admitted to the hospital on Wednesday, with the ordinary symptoms of stone. On Thursday, was brought before the Class, and the sound revealed the existence of a stone of an inch or more in diameter. On Friday, was again brought before the Class for operation. The lithotribe causing considerable pain, he was given chloroform. The stone was with some difficulty seized, and a part of it crushed, some of the fragments being brought or washed away. For some reason, the operation was not completed, and the patient returned to his bed. In the course of twenty-four hours he was suffering intense pain, which soon increased to almost a mania, and Saturday evening he died.

*Post mortem* revealed cystitis, pericystitis, and peritonitis, to an extreme degree, with a perforation of the bladder posterior to the prostate. No fragment was found outside the bladder; and whether the perforation was made by the stone or instrument, was not decided.

I've seen this operation several times here, but none of lithotomy. F.

